# Geographic Determinants of Healthy Lifestyle Change in a Community-Based Exercise Prescription Delivered in Family Practice

**DOI:** 10.4137/EHI.S820

**Published:** 2008-10-01

**Authors:** Robert J. Petrella, Emily Kennedy, Tom J. Overend

**Affiliations:** Faculties of Medicine and Health Sciences, University of Western Ontario, London, Ontario, Canada

## Abstract

**Background:**

Evidence is unequivocal that exercise training can improve health outcomes. However, despite this evidence, adoption of healthy lifestyles is poor. The physical environment is one possible determinant of successful adoption of healthy lifestyles that could influence outcomes in community-based intervention strategies. We developed a novel exercise prescription delivered in two different cohorts of older sedentary adults—one delivered by family physicians to patients with identified cardiovascular risk factors (CRF) and the other delivered at a community exercise facility to a larger cohort of healthy sedentary adults (HSA). We then determined whether the place of residence and proximity to facilities promoting physical activity and healthy or unhealthy eating could influence clinical changes related to these community-based exercise prescriptions.

**Methods:**

Two different cohorts of older patients were administered similar exercise prescriptions. The CRF cohort was a sedentary group of 41 older adults with either high-normal blood pressure (120–139 mmHg/85–89 mmHg) or impaired glucose tolerance (fasting glucose 6.1–6.9 mmol/l) who were prescribed exercise by their family physicians at baseline and followed over 12 months. The HSA cohort consisted of 159 sedentary older adults who were prescribed a similar exercise prescription and then participated in a chronic training program over 5 years at a community-based training facility. Outcomes of interest were change in fitness (VO_2max_), resting systolic blood pressure (rSBP) and body mass index (BMI). GIS-determined shortest distance to local facilities promoting physical activity and healthy versus unhealthy were compared at baseline and followup using simple logistic regression.

Those subjects in CRF group were further identified as responders (exhibited an above average change in VO_2max_) and were then compared to non-responders according to their patterns of proximity to physical activity and eating facilities.

**Results:**

In the CRF cohort at baseline, greater GIS-distance to golf courses correlated with higher rSBP (r = 0.38, p = 0.02) while greater distance to bike paths correlated with greater BMI (r = 0.32, p = 0.05). CRF responders who lived closer to a park had higher BMI (r = −0.46, p = 0.05) while no other relationship among responders and proximity to either physical activity or eating facilities was observed. CRF non-responders lived closer to formal physical activity facilities (community centres) and higher fat eating facilities. In the HSA cohort, higher fitness was correlated with greater distance to both formal and informal physical activity facilities (baseball fields or dance studios) while this was also correlated with a higher rSBP (r = 0.17, p = 0.04). In general, physical activity facilities were often located near higher-fat eating facilities regardless of cohort.

**Conclusion:**

Those prescribed exercise by their family physician for the presence of health risk tended to closer to any type of physical activity facility compared to those who joined an exercise program on their own. A positive response to the intervention at 12 months was associated with closer access to informal physical activity facilities while non-responders lived closer to both types of physical activity facility as well as high fat eating facilities. In contrast, healthy chronic exercise trainees in the community did not show any meaningful relation between fitness and proximity to healthy lifestyle facilities. Hence, the access to facilities is not as important to those who adopt physical activity on their own whereas those targeted by physicians may be influenced by access. Furthermore, the response or lack thereof to exercise interventions in those at risk may be influenced by proximity to both physical activity and unhealthy eating facilities.

## Introduction

With demographic trends of an aging, sedentary society leading to epidemics of obesity and cardiovascular disease (CVD), there is a need to expand preventative health care measures into the community infrastructure. The significance of creating physical environments that are supportive of healthy lifestyles received formal recognition in the 1986 Ottawa Charter for Health Promotion.^1^ Physical environmental factors affecting sedentary behaviour and health include actual and perceived access to physical activity facilities, regional aspects such as urban location, presence of enjoyable scenery and terrain, the climate or season of the year, heavy traffic as well as more subtle neighbourhood characteristics.^5^,^6^,^7^ At the more intimate, neighbourhood level, quantitative insight can be obtained regarding access to specific facilities including cycle paths,^8^ parks,^9^ drive-by physical activity facilities on frequently traveled routes,^10^ density of pay or free facilities^11^ and access within walking distance.^6^ Although intuitively, the access to healthy versus unhealthy eating facilities including location and density would seem to provide a similar health relationship as access to physical activity facilities, this has not been investigated to the same extent^19^ nor has the interaction of physical activity and healthy eating facilities been investigated in relation to the presence of CVD risk factors^20^ or whether the response of patients to structured interventions as opposed to self-selection, can be associated with the physical environment in which the interventions are prescribed.

Recommendations regarding prescription of exercise^2^ and dietary^10^ change, while widely disseminated to the public, have not had a significant impact on a change in lifestyle behaviours.^11^,^12^,^13^,^21^ Hence, efforts have more recently been directed at the delivery of exercise and dietary interventions in the primary care setting^22^–^25^ to identify specific issues of behaviour change with exercise counselling and prescription.^18^,^26^ However, clinical trials of lifestyle intervention have not investigated or implicated the role of geographic/physical environmental correlates with observed change in health. Hence, we were interested in exploring the relationship between geographic/physical environmental attributes, specifically, distance to facilities promoting physical activity and higher versus lower fat eating with indices of cardiovascular health including fitness (VO_2max_), systolic blood pressure (SBP) and body mass index (BMI) in two representative cohorts of older adults: one with CVD risk factors who were prescribed exercise by their family physician, and another who were regular long-term participants in a community-based exercise program.

## Methods

To determine the environmental effects of proximity to physical activity and healthy eating facilities on health outcomes including VO_2max_, SBP and BMI, we compared two distinct cohorts of London, Ontario residents with geographic profiling using ArcView 8.3 (ESRI, Redlands, 2002). The primary outcome variable was the average shortest GIS-distance (km) to physical activity and eating facilities. Fitness (ml O_2_ · kg^−1^· min^−1^), SBP (mmHg), and BMI (kg/m^2^) were analyzed for their relationship to the primary outcome. GIS data were obtained from the map libraries of the Department of Geography at the University of Western Ontario and the City of London.

## Subjects

Subject data were obtained from two distinct cohorts from London, Ontario (population 300,000): a group of older patients with identified CVD risk factors (CRF) who were enrolled in a 12 month family physician-delivered exercise prescription trial,^18^ and another group of older healthy sedentary adults (HSA) who voluntarily joined and then regularly attended a community-based exercise training program over 7 years.^26^ Both cohorts have been previously described.^26^ Both groups were prescribed similar training as per published recommendations.^2^

The CRF group represented forty-one subjects from the city of London who were part of a larger randomized trial of exercise prescription (STEP) among 48 family physicians across Canada and 480 of their older sedentary patients.^18^,^19^ Patients were prescribed exercise using a novel instrument and followed over 12 months.^18^

The HSA cohort included a random sample of 159 subjects from a total registry of 684 older adults (45% male) aged 55–75 years old who had attended regular exercise sessions (30–45 minutes) of aerobic activity (walking, jogging) three times per week in a community exercise program at the Canadian Centre for Activity and Aging for at least 5 years at the time of this study.^19^

## Cardiovascular Risk Factor Measures

Determination of VO_2max_ was conducted by a trained kinesiologist using a modified Naughton treadmill protocol.^18^ Blood pressure was obtained in the seated position following 5 minutes rest using an automated oscillometric device (BPTru, VSM MedTech, Vancouver). Body mass index was calculated as weight divided by the square of height (kg/m^2^).

## GIS Environmental Measures

**Figure 1.**
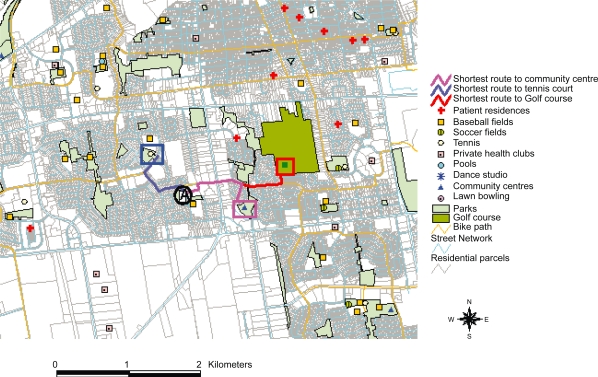
Example of road network analysis using arcview 3.2.

Traditional methods of measuring geographic correlates of healthy behaviour have utilized qualitative survey data such as perceived access to physical activity facilties. While informative in identifying associations, quantitative data for sophisticated geographic, behavioural and health modelling are needed. Geographic Information Systems (GIS) are a novel quantitative approach used to study healthy lifestyle attributes as they relate to the built environment^36^ allowing for the integration and analysis of geographic data (i.e. shortest distance) through mapping specific areas and attributes with resultant high reliability of the data^17^,^35^ (Fig. 1).

Physical activity facilities were classified as providing “formal” and “informal” opportunities for healthy behaviour (Table 1). Formal opportunities were defined as requiring a regular paid membership or “pay-as-you-go” user fees, requiring reservations or scheduled times for use and/or requiring participation on organized teams. Informal opportunities required no payment and were available to any member of the public without reservations or participation as part of a team.

Healthy or unhealthy eating facilities (Table 2) were classified on two levels by a trained nutritionist. First, by gross inspection, they were defined as “fast food” or “dine-in” according to the North America Industry Classification System (NAICS) whereby fast-food or limited-service restaurants were establishments where patrons would order or select items for quick consumption or take away. Dine-in or full-service restaurants were defined as providing food services to patrons who would order and be served while seated (i.e. waiter/waitress service), pay after eating and consisted of more than one course. Our assumption that the latter establishments would provide more healthful options was confirmed using a second classification. The well described threshold of dietary fat for prevention of diet-related heart health is consumption less than 30% of total energy. This second classification included abstracting menus from all eating establishments within London, Ontario using business and telephone directories and field visits. Hence, eating establishments were further classified as “higher fat” or “lower fat” based on the proportion of menu items that contained more than 30% of energy from fat. If over two-thirds of a restaurant’s menu items contained over 30% fat (e.g. deep fried foods, hamburgers) the restaurant was classified as “higher fat”.

Geocoding of subjects’ home addresses, physical activity and eating facilities was done using the interactive CityMap on the City of London website (www.london.on.ca) and the road network in ArcMap™ 8.3 (ESRI, Redlands, 2002). Network analysis was performed to determine the shortest distance from subjects’ home addresses to physical activity and eating facilities. Description of the process of performing the primary analysis as given in Table 3.

## Analysis

All data analysis was done using SPSS Student Version 11.0 for Windows (SPSS Inc., 2003). Univariate logistical regression models were conducted with shortest GIS-distance to physical activity and then eating facilities (independent variables). Baseline and post training relationships were determined in both cohorts. In the CRF and HSA cohorts, post 12 month and 5-year data were grouped according to whether the subjects showed a greater than group mean change from baseline in VO_2max_ (response) versus non-response. Responders were plotted against non-responders to determine if response was associated with proximity to geographic determinants. Student’s *t*-tests were utilized for comparison between responder and non-responder groups. All values were reported as means ± SD and significance was defined as p < 0.05.

## Results

### Subject characteristics

Subjects from the CRF cohort (n = 41) included 24 males and 17 females aged 52 to 79 years (mean 64 ± 8.4 years). At baseline, the mean VO_2max_ was 28.2 ± 7.4 O_2_ ml kg^−1^ · min^−1^, mean SBP was 134 ± 16 mmHg and BMI was 29.4 ± 6.2 kg·m^−2^ (Table 4).

After twelve months, twenty-one STEP subjects were considered responders to the intervention. At baseline, responders versus non-responders had similar mean VO_2max_ (33.1 ± 6.0 ml O_2_ kg^−1^ min^−1^ versus 30.4 ± 8.5 ml O_2_ kg^−1^ min^−1^), mean SBP (136.5 ± 17.2 mmHg versus 138.5 ± 15.3 mmHg) and BMI (27.7 ± 10.2 kg·m^−2^ versus 27.9± 6.9 kg·m^−2^). After 12 months, responders showed an increase of 3.3 ± 1.7 ml O_2_ kg^−1^ · min^−1^ in VO_2max_ (p = 0.001), an increase of 2.1 ± 18.5 mmHg in rSBP (p = 0.08), and a decrease of 0.07 ± 4.0 kg·m^−2^ in BMI (p = 0.001).

The HSA cohort included 72 males and 87 females aged 48 to 92 years (73 ± 8.8 years). The mean VO_2max_ was 28.4 ± 9.1 O_2_ ml kg^−1^·min^−1^, SBP was 133.7 ± 14.1 mmHg and BMI was 26.5 ± 4.7 kg·m^−2^. All subjects improved VO_2max_ at followup so no further analysis of response was conducted in this group. Aside from being older, there were no differences between groups at baseline. Of note, both cohorts met the criteria for having high-normal blood pressure and being overweight.

## Environmental Characteristics of the Population

The City of London was divided into North, South and East regions based on the divisions of the London Real Estate Board that uses the natural boundary of the Thames River. The CRF subjects (n = 159) distributed homogeneously while the HSA subjects (n = 41) were more widely distributed. Specifically, 80% percent of HSA subjects (n = 128) lived in the North, while 14% (n = 23) lived in the East and 5% (n = 8) lived in the South. Thirty-four percent of HSA subjects (n = 14) lived in the North, while 20% (n = 8) lived in the East and 46% (n = 19) lived in the South.

### Environmental attributes

**Figure 2.**
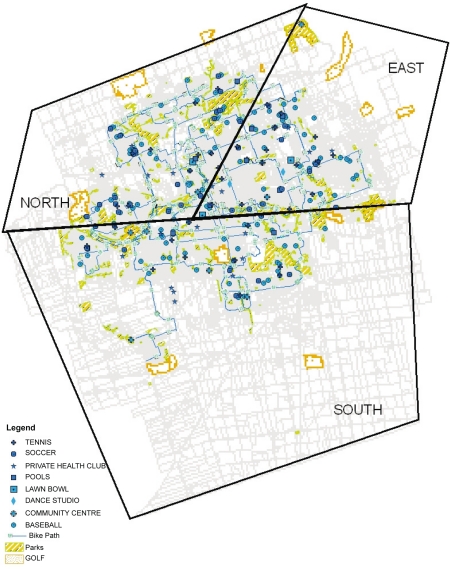
Geographic distribution of physical activity facilities.

The 253 formal opportunities for PA were distributed throughout the city (Fig. 2). For informal opportunities, over 40% of the 280 public parks were located in the South region, about 30% were in the North and about 20% were in the East. There were 296 segments of multiuse and bike paths throughout the city and almost 40% were in the North region. Figure 2 illustrates distribution of facilities.

**Figure 3.**
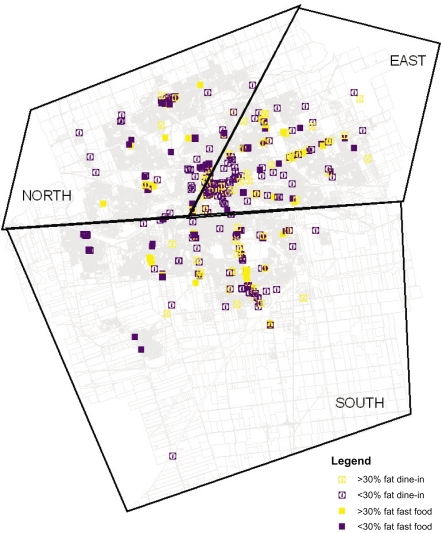
Geographic distribution of eating opportunities. Greater than 2/3 of a restaurant’s menu items >30% fat (>30% fat); Less than 2/3 of a restaurant’s menu items >30% fat (<30% fat).

There were 132 fast-food restaurants and 335 dine-in restaurants. Dine-in restaurants out-numbered fast-food restaurants in each region. Over 70% of the fast-food restaurants were in the South or East regions of the city while dine-in restaurants were mostly in the East region. For all regions, lower-fat fast-food restaurants such as Subway and pizza outlets out-numbered high-fat fast-food restaurants like Kentucky Fried Chicken and burger outlets. Lower-fat dine-in restaurants such as East Side Mario’s and deli eateries also out-numbered high-fat dine-in restaurants like Kelsey’s and steakhouses in all regions. Over 60% of high-fat bar and grill restaurants were located in the East region. Over 85% of lower-fat deli and soup ‘n’ sandwich cafés were found in the East and North regions. Figure 3 illustrates the geographic distribution of all eating opportunities.

#### GIS distance outcomes

For the HSA cohort, the mean distance to the closest PA facility was 1.83 ± 1.06 kilometers (km) and ranged from zero kilometers to the closest public park to 9.38 km to the closest lawn bowling facility. The closest informal PA facility averaged 0.37 ± 0.38 km away (range zero to 3.26 km). Unfortunately, distance to the closest bike/multi-use path was not evaluated because access point locations could not be obtained. Average distance to the closest formal PA facility was 3.28 ± 1.73 km and ranged from a minimum of 0.02 km to the closest private health club to a maximum of 9.38 km to the closest lawn bowling facility. Hence, informal PA facilities were, on average, closer than formal PA facilities.

Eating opportunities averaged 1.49 ± 1.10 km to the closest fast food or dine-in restaurant. The mean distance to the closest fast food outlet was 1.35 ± 0.85 km (range 0.16km to 5.43 km). The mean drive time to the closest fast food outlet was 2.70 ± 1.35 minutes (range 0 to 9 min). Average distance to the closest dine-in restaurant was 1.63 ± 1.35 km (range 0.08 km to 6.63 km). Average drive time to the closest dine-in restaurant was 2.88 ± 1.95 min and, similar to the drive time to the closest fast food outlets (range 0 to 9 min). Higher fat (greater than 30%) fast food outlets were closer than lower fat (less than 30%) fast food outlets, averaging 1.67 ± 1.06 km versus 1.49 ± 0.80 km, respectively. In contrast, higher fat dine-in restaurants were, on average, further away than lower fat restaurants (2.45 ± 1.64 km versus 1.86 ± 1.63 km, respectively).

In the CRF cohort, the mean distance to the closest PA facility was 3.82 ± 0.99 km. Again, public parks were the closest out of the all PA facilities at a minimum of 0.01 km from a CRF subject while dance studios were the furthest at a maximum of 8.95 km from a CRF subject. The closest informal PA facility (public park) averaged 0.5 ± 0.41 km away (range 0.01 km to 1.29 km). Average distance to the closest formal PA facility was 4.24 ± 1.07 km ranged from 0.04 km to the closest golf course to 8.95 km to the closest dance studio. Once again, informal PA facilities were closer than formal PA facilities.

Among all eating opportunities, the mean distance was 1.32 ± 1.51 km. Average distance to the closest fast food outlet was 1.38 ± 1.53 km (range 0.22 km to 10 km). The mean drive time to the closest fast food outlet was 2.51 ± 2.39 min and ranged from 1 to 16 min. Average distance to the closest dine-in restaurant was 1.28 ± 1.48 km, ranging from 0.13 to 6.09 km. Average drive time to the closest dine-in restaurant was 2.20 ± 1.91 minutes and ranged from 0 to 9 minutes. Higher fat and lower fat fast food outlets had very similarly average distances and minimum distances (1.52 ± 1.56 km versus 1.50 ± 0.92, respectively and 0.22 km). However, lower fat fast food outlets had a wider range of distances and could be up to 10 km from a STEP subject while the higher fat option had a maximum distance of only 4.28 km. The two types of dine-in restaurants were also fairly similar average distances and range for this cohort, 1.41 ± 1.65 km to the closest lower fat option and 1.69 ± 1.3 7 km to the closest higher fat dine-in restaurant.

Responders to the intervention among CRF subjects averaged 4.99 ± 1.87 km to the closest PA facility versus non-responders who averaged 5.37 ± 2.11 km to the closest PA facility. Public parks were the closest PA facility for both groups, while tennis courts were the greatest distance from responders and soccer fields were the furthest away for non-responders. Informal PA facilities ranged from 0.01 to 1.04 km from a responder versus 0.05 to 0.93 km from a non-responder. Formal PA facilities ranged from 0 to 19.35 km from a responder versus 0.5 to 18.37 km from a non-responder. Overall, though not statistically significant so, responders were closer to both informal and formal PA facilities than non-responders.

With respect to eating, responders averaged 1.32 ± 1.14 km to the closest EO versus non-responders were slightly further at an average of 1.47 ± 2.44 km. The closest fast food outlet was an average of 1.23 ± 0.66 km from a responder versus an average of 1.34 ± 0.67 km away from a non-responder. Similarly, responders were slightly closer to dine-in restaurants (M = 1.41, SD = 1.61) as compared to non-responders (M = 1.60, SD = 1.77). In both groups, lower fat eating opportunities were closer than high fat eating opportunities. Table 5 summarizes the distance to PA facilities and eating opportunities from both cohorts’ place of residence.

## Geographic Interactions

In the CRF cohort at baseline, higher SBP (r = 0.38, p = 0.02) and BMI (r = 0.32, p = 0.05). were significantly correlated with greater GIS-distance to some formal and informal physical activity facilities (golf courses and bike paths specifically). Non-responders showed shorter GIS-distance to formal physical activity facilities including community centers, dance studios, lawn bowls and swimming pools that also correlated with shorter GIS-distance to high fat eating facilities (i.e. fast food, dine-in and higher low fat). In comparison, the responders (p = 0.001) demonstrated that closer proximity to private health clubs was significantly associated with closer proximity to higher fat and fast food establishments (r = 0.53, p = 0.04). Neither responders nor non-responders showed any correlation with SBP and distance to physical activity facilities. However, greater change in BMI (reduced) in responders was correlated with shorter distance to parks (r = −0.46, p = 0.05).

In the HSA cohort, higher VO_2max_ was correlated with greater GIS-distance to some formal and informal physical activity opportunities (dance studios (r = 0.18, p = 0.03) and baseball diamonds (r = 0.09, p = 0.04)). These facilities were also more distant from subjects having higher SBP (baseball diamonds (r = 0.17, p = 0.04) and dance studios (r = 0.17, p = 0.04). Greater distance to dance studios was correlated with higher BMI (r = 0.19, p = 0.02). Regarding eating establishments, higher SBP was significantly correlated with greater distance to higher fat establishments (r = 0.16, p = 0.05).

In general, any type of physical activity facility was located near higher fat eating facilities.

## Discussion

While effective in clinical trials, broader community-delivery of lifestyle interventions have not been adopted or achieved the same impact.^2^,^15^,^16^ The influence of the physical environment may be an important determinant of the success of healthy individuals to maintain, or those with risk factors to improve their healthy physical activity and eating,^33^ others habits. However, data regarding the relationship between healthy lifestyle facilities and indicators of healthy lifestyle are limited.^13^,^16^

The primary objective of this study was to investigate the association and relationship between the physical environment, defined as access to healthy physical activity and eating facilities and fitness, blood pressure and BMI in two distinct but representative cohorts of older adults. Specifically, one cohort (CRF) represented those with early risk factors enrolled in a lifestyle intervention delivered by their family physicians, while the other (HSA) were regular participants in a community exercise program interested in maintenance of healthy lifestyle habits.

The CRF cohort showed higher blood pressure and lower levels of baseline fitness with greater GIS-distance to either formal or informal physical activity facilities. At 12 months post-intervention, non-responders were more likely to live closer to either physical activity and higher fat eating facilities compared to responders who showed no relationship with proximity to physical activity facilities. The HSA cohort showed that higher fitness was correlated with greater proximity to either formal and informal physical activity facilities. Proximity to unhealthy eating facilities in the CRF non-responders was correlated with closer proximity to formal physical activity facilities while responders did not show any relationship between physical activity facilities and eating establishments. Conversely, the HSA cohort showed higher blood pressure with closer proximity to higher fat establishments regardless of proximity to either type of physical activity facilities.

The positive effect in our study of proximity to some but not all physical activity facilities on health predictors such as BMI has been described^19^ and could reflect a preference for certain activities including golf or tennis over lawn bowling or swimming reflected as choices of place of residence in the study population. The HSA cohort in our study has been previously described^31^ as having a more heterogeneous place of residence than the CRF group reflecting choices (or not) in supporting a healthy lifestyle. Since homogeneous study populations may have been a limitation in previous studies^32^ the inclusion of two different groups of subjects may have minimized this effect in our study. Further, the CRF group may provide some insight regarding possible determinants of successful adoption of exercise prescriptions by health professionals based on homogeneous distribution of residence as well as distribution of related health outcomes and environmental correlates.

It is difficult to compare the results of the present study with previous studies since while qualitative demographic characteristics^4^,^33^,^34^ have been described, quantitative physiological measures (fitness and blood pressure for example) and GIS methodology have not been reported. Also, the impact of the physical environment on adherence to a lifestyle intervention has not been previously described.

In the CRF cohort, shorter distance to golf courses was correlated with lower SBP. While there are no published studies on distance to golf courses and blood pressure, Parkkari et al. studied the positive effects of golf on fitness and body composition in a controlled clinical trial.^30^ A further search for the interaction of blood pressure and the built or physical environment including physical activity facilities or healthy eating establishments revealed an absence of data. Hence, our findings are unique and should be studied further in the context of physical activity choices, access in the community and the association with health outcomes. In particular, the development of healthy living communities should consider the access to and types of physical activity and eating establishments available to populations who will live there, while existing communities should consider promotion, development and access to healthy lifestyle establishments as a prevention strategy across all populations they serve.

Neither classification of eating establishments had an influence on the effect of proximity on fitness or BMI in either cohort although non-responders did live closer to higher fat eating facilities. Only SBP was significantly associated with the type of eating establishment in the HSA cohort. Specifically, proximity to lower fat and dine-in restaurants were associated with lower SBP which is in agreement with previous reports of a link between low fat and lower blood pressure^27^,^28^ and improved cardiovascular health.^29^ However, the association between distance to lower fat and dine-in restaurants and CVD risk factors has not been previously reported until the current study and should be studied further.

Given the close interaction between quality and quantity of activity and diet for achievement of a healthy lifestyle, the concomitant proximity of complementary or physical activity or eating facilities is of importance to public health policy and planning. In particular, we observed that closer proximity to most physical activity facilities was associated with closer proximity to fast food outlets in both the HSA cohort, where this did not appear to influence the impact of participating in a community-based exercise program, but also in the CRF non-responders, where the easy access to unhealthy eating establishments may have posed a barrier to increasing activity despite close access to these facilities. Further, responders, living closer to fast food outlets did not correlate with living closer to physical activity facilities. This may suggest that these individuals were able to overcome dietary challenges in their community towards improving fitness and health. Increasing fitness, either among those who have adopted chronic exercise on their own, or those targeted by health professionals, may have other behavioural attributes that leverage the success of the environment and interventions. The scenario of “if you build it they will come” may indeed be “will they come because you built it?”. Regardless, this preliminary study suggests that future investigations of lifestyle and healthy environments should consider the behavioural change determinants of individuals when interpreting or translating findings into practice and policy change.

## Conclusion

This study investigated the interaction of physical environment defined as healthy physical activity and eating facilities, with indicators of health in two cohorts of older adults differing in physical activity habits and cardiovascular risk. The built environment, in particular access to unhealthy eating establishments, may not impart the same impact on health among those who successfully adopt healthy physical activity either in the short term or chronically. It is also interesting that those who were prescribed exercise and successfully improved their fitness appeared to do so with some association to access of formal as opposed to informal physical activity facilities. Those who did not improve fitness with this intervention may have succumbed to the closer access of unhealthy eating establishments despite access to formal physical activity facilities. A further study will determine if these findings are related to perceived or actual use of the physical activity and eating facilities in these cohorts. Further study of behaviour as well as other modifiable factors influencing physical activity will clarify questions regarding the prediction of a response to lifestyle interventions which can be helpful to health providers, the public and urban planning policy makers. These studies should be considered in the context of multi-sectoral collaboration to “build” healthy communities.

## Figures and Tables

**Table 1.** Classification of physical activity facilities.

	**Opportunity for PA**	**Example**
**Formal**	Private health clubs	Goodlife Fitness Clubs
	Golf courses	London Hunt and Country Club
	Community centres	Hamilton Road Senior’s Centre
	Tennis courts	A.B. Lucas Secondary School Tennis Courts
	Swimming pools (public)	London Aquatic Centre Indoor Pool
	Lawn bowls	Elmwood Lawn Bowling Club
	Skating Arenas	Argyle Arena
	Dance studios	Fred Astaire Dance Studio
	Baseball fields	Thames Park Baseball Diamond
	Soccer fields	Byron Optimist Community Centre Soccer Fields
**Informal**	Public parks	Gibbons Park
	Bike paths	Various on-street and multiuse paths throughout city
	Walking trails	Downtown Discovery Trail

**Table 2.** Classification of eating opportunities.

**Primary class**	**Secondary class**	**Examples**
Fast-food	Low fat	Mr. Sub, Domino’s Pizza, Tim Hortons
	High fat	McDonald’s, KFC, Taco Bell
Dine-in	Low fat	Mount Fuji Japanese Restaurant, Mexicali Rosa’s, Bertoldi’s Trattoria
	High fat	Kelsey’s Restaurant, The Keg, Archie’s Seafood Restaurant

**Table 3.** 

**Primary analysis**	**Description**
Themes	Digitized ‘themes’ or ‘layers’ were obtained from the City of London. These themes included a road network (map of local streets), bike paths, multiuse pathways and parks.
Geocoding	The place of residence (origin) for each participant was ‘address matched’ (geocoded) using the GIS software. At a basic level this involves identifying and labeling each participant’s address on the digitized street map. Any additional ‘destinations’ that were not available in ‘themes’ from the City of London were also be address matched at this step.
Spatial Database Management	Once geocoding was complete attributes were attached to each ‘origin’. These attributes were obtained using CRF and the HSA data that was transposed into a usable form for GIS software.
Road Network Analysis	To determine the distance between origins (participant residences) and the following destinations based on the shortest route to all physical actiivity and eating opportunities. (See Fig. 1)

**Table 4.** Physiological statistics of CRF subjects.

	**Population**	**Responders**	**Non-Responders**	***p* value**
N	41	21	20	
*At baseline*:				
VO_2max_ (ml O_2_ kg^−1^ · min^−1^)	28.2 ± 7.4	33.1 ± 6.0	30.4 ± 8.5	0.07
SBP (mmHg)	133.9 ± 15.8	136.5 ± 17.2	138.5 ± 15.3	0.10
BMI (kg/m^2^)	29.4 ± 6.2	27.7 ± 10.2	27.9 ± 6.9	0.17
*After 12 months*:				
ΔVO_2max_ (ml O_2_ kg^−1^·min^−1^)	+1.2 ±3.1	+ 3.3 ± 1.7	−1.8 ± 1.9	0.001
ΔSBP (mmHg)	+3.2 ± 16.2	+2.1 ± 18.5	+3.9 ± 17.5	0.03
ΔBMI (kg/m^2^)	+0.17 ± 2.6	−0.07 ± 4.0	+0.3 ± 1.9	0.001

Resting Systolic Blood Pressure (SBP); Body Mass Index (BMI); Responders were subjects with a greater-than-group-average change in VO_2max_ while non-responders had a change in VO_2max_ less than the group average; VO_2max_, SBP and BMI values are means ± SD.

**Table 5.** Distance to physical activity facilities and eating opportunities.

	**HSA**	**CRF**	
		**Responders**	**Non-responders**
**PA Facilities**	1.83 ±1.06	4.99 ±1.87	5.37 ± 2.11
Formal (range)	3.28 ±1.73 (0.02 to 9.38)	6.65 ±3.51 (0 to19.35)	6.68 ± 3.47 (0.5 to 18.37)
Informal (range)	0.37 ±0.38 (0 to 3.26)	0.39 ±0.27 (0.01 to 1.04)	0.42 ± 0.25 (0.05 to 0.93)
**Eating Opportunities**	1.49 ± 1.10	1.32 ±1.14	1.47 ± 2.44
Fast Food (range)	1.35 ± 0.85 (0.16 to 5.43)	1.23 ± 0.66 (0.23 to 2.83)	1.34 ± 0.67 (0.13 to 6.09)
Higher fat	1.67 ± 1.06	1.69 ± 0.97	1.85 ± 1.05
Lower fat	1.49 ± 0.80	1.35 ± 0.72	1.48 ± 0.73
Dine-in (range)	2.70 ±1.35 (0.08 to 6.63)	1.41 ± 1.61 (0.13 to 6.09)	1.6 ± 1.77 (0.18 to 6.09)
Higher fat	2.45 ± 1.64	1.98 ± 1.51	1.97 ± 1.56
Lower fat	1.86 ± 1.63	1.56 ± 1.84	1.79 ± 2.01

Physical Activity (PA); HSA cohort; Responders were subjects with a greater-than-group-average change in VO_2max_ while non-responders were CRF subjects with a change in VO_2max_ less than the group average; All values were in kilometers and expressed as means ± SD.
